# Analysis of the epidemiological situation of influenza in Guangzhou under the prevention and control of COVID‐19 in June 2022

**DOI:** 10.1111/crj.13754

**Published:** 2024-05-01

**Authors:** Yuezhi Deng, Yongping Lin, Weihong Lin

**Affiliations:** ^1^ Department of Laboratory Medicine, The Key Laboratory of Advanced Interdisciplinary Studies Center The First Affiliated Hospital of Guangzhou Medical University, National Center for Respiratory Medicine, National Clinical Research Center for Respiratory Disease Guangzhou China; ^2^ National Cancer Center/National Clinical Research Center for Cancer/Cancer Hospital & Shenzhen Hospital, Chinese Academy of Medical Sciences and Peking Union Medical College Shenzhen China

**Keywords:** COVID‐19, epidemiological situation, fever, Guangzhou, influenza

## Abstract

**Objective:**

Under the prevention and control measures of COVID‐19, the epidemiological situation of respiratory pathogens is not well known. Understanding the patterns of respiratory pathogens epidemiology under the prevention and control measures of COVID‐19 is important to guide resource allocation for existing and future treatment and prevention strategies.

**Methods:**

In total, 659 fever outpatients nasopharyngeal swabs were collected at fever illness onset during June in 2022 at the First Hospital of Guangzhou Medical University. Swabs were tested by real‐time fluorescent single‐tube multiplex polymerase chain reaction (PCR) for 12 respiratory pathogens. Moreover, 108 of the 659 swabs were tested for influenza virus antigen.

**Results:**

At least one pathogen was detected in 477 (72.38%) of 659 fever outpatients with multiple pathogens identified in 25 (3.79%). The highest multiple infectious pattern is parainfluenza virus in combination with influenza (five cases). Influenza A virus (IFA), human rhinovirus (HRV), and parainfluenza virus are the three leading virus pathogens with proportions of 64.64%, 5.01%, and 2.88%. School‐age children and adult groups have the highest pathogens positivity rate of 81.28% and 83.87%.

**Conclusion:**

A high proportion of adolescents and adults has respiratory pathogens detected during fever illnesses during June in 2022 under the prevention and control of COVID‐19. These data indicate that diagnosis, prevention, and control of respiratory tract infection should be paid attention under the prevention and control of COVID‐19.

## Graphical abstract

In this work, Multiplex Probe Amplification (MPA) technique was used to detect 12 respiratory viruses in the nasopharyngeal swabs of 659 fever outpatients, showing a relatively high positive rate (72.38%), including multiple infections (3.79%). The positive rate of influenza A (64.64%), and infection was the highest, followed by HRV (5.01%) and PIV (2.88%). A high proportion of adolescents and adults has respiratory pathogens detected during fever illnesses during June in 2022 under the prevention and control of COVID‐19.

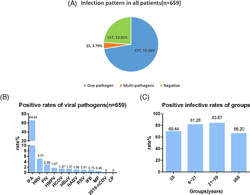

## 1 INTRODUCTION

Acute respiratory infection (ARI) is a significant global health concern characterized by high morbidity and mortality rates.^1^ Conditions such as the common cold, viral pharyngitis, bronchitis, and pneumonia all fall under this category.^2^, ^3^, ^4^, ^5^ According to the World Health Organization, lower respiratory tract infections rank as the fourth leading cause of death worldwide. Annually, influenza infections account for 1 billion cases globally, with 3–5 million being severe cases.^6^ Acute lower respiratory tract infections, including pneumonia and bronchitis, are the primary causes of hospital‐related deaths among young children, particularly in low‐ and middle‐income countries.^7^ Pneumonia resulting from acute lower respiratory tract infections is also a leading cause of death among children under 5 years old in China.^8^

The pathogens responsible for respiratory tract infections are diverse, including bacteria, viruses, and other atypical agents. Identifying the causative pathogen aids in diagnosis and guides the appropriate use of antimicrobial drugs.^2^, ^3^, ^4^, ^5^ However, viral infections often lack targeted treatments and have fewer diagnostic tests available. Historically, some clinicians considered viral treatment to have limited clinical value because of the absence of specific antiviral drugs, leading to a neglect of testing for respiratory viruses. The COVID‐19 pandemic has underscored the importance of viral testing not only for clinical prognosis but also for implementing effective hospital infection control measures, breaking transmission chains, and preventing the spread of the virus.^9^

Influenza is one of the most common pathogens causing acute upper respiratory tract infections. The primary symptoms of influenza include a sudden onset of fever (body temperature ≥ 38.0°C) accompanied by cough and body aches. The influenza viruses are mainly transmitted via respiratory droplets and are highly contagious. In China, influenza vaccination rates and willingness to vaccinate are generally low, and reports of oseltamivir resistance have increased the burden on influenza prevention and control efforts.^10^, ^11^, ^12^, ^13^ After the outbreak of COVID‐19, some studies show a significant decrease in the prevalence of influenza and pneumonia.^14^, ^15^ However, our hospital in Guangzhou has observed a noticeable uptick in fever clinic visits since the beginning of June 2022. To enhance diagnostic accuracy, reduce medication overuse, and prevent viral outbreaks, we analyzed the epidemiological situation of influenza in Guangzhou for June 2022. Our study found that influenza A virus was the predominant upper respiratory tract pathogen in Guangzhou during that month, significantly outnumbering other pathogens.

## 2 SUBJECTS AND METHODS

### 2.1 Subjects

A total of 659 fever (≥37.3°C) outpatient specimens were randomly collected from patients at the fever outpatient clinic of the First Affiliated Hospital of Guangzhou Medical University in June 2022.

### 2.2 Sample preparation and nucleic acid extraction

The specimens were brought to room temperature, and the swabs were thoroughly mixed using a shaker. The swabs were then repeatedly squeezed against the tube wall to remove excess liquid. The liquid was centrifuged at 4°C for 10 min at 3000 rpm, after which the supernatant was removed, leaving the precipitate ready for testing. Nucleic acids were extracted using a fully automated nucleic acid extractor (Daan Swift96, Guangzhou Da'an Gene Co., Ltd., Guangzhou, China) following the manufacturer's instructions. The extracted nucleic acids were stored at −80°C for future use.

### 2.3 Multiplex probe amplification (MPA)

The extracted nucleic acids were detected by multiplex polymerase chain reaction (PCR) using a SLAN 96P fluorescent quantitative PCR instrument (Shanghai Hongshi Medical Technology Co., Ltd., Shanghai, China). The commercial reagent kits were purchased from Guangzhou Biotron Technology Co., Ltd. (Guangzhou, China). The kit name and catalog number are as follows: “Respiratory Pathogens Twelve‐item Joint Test Kit (PCR‐Chromatography Strip Hybridization Assay)” with catalog number BB5013. These kits included reagents for detecting a variety of pathogens such as Chlamydia pneumonia, Mycoplasma pneumonia, adenovirus (Group B, Group C, Group E), influenza A virus (H1N1, H3N2, H1N1 [2009], H5N1, H7N9), human influenza B virus (Victoria, Yamagata), parainfluenza virus (types 1, 2, 3, 4), rhinovirus, respiratory syncytial virus (types A and B), bocavirus, metapneumovirus, coronavirus (229E, HKU1, NL63, OC43), ORF1ab gene of COVID‐19, and N gene of COVID‐19. For certain pathogens like adenovirus, influenza A and B viruses, parainfluenza virus, respiratory syncytial virus, and coronavirus, subtyping was not performed.

### 2.4 Sanger sequencing and comparative analysis

The extracted nucleic acids underwent reverse transcription and sequencing amplification. Sequencing was carried out by the Guangzhou branch of Beijing Liuhe Huada Gene Technology Co., Ltd. (Guangzhou, China). The peak maps were assembled using SeqMan software, and the sequencing results were compared and identified using BLAST in the NCBI database. The primers are shown in Table S1.

### 2.5 Antigen reagent detection

Influenza A/B virus antigen kit (colloidal gold method, Nanjing Synthgene Medical Technology Co., Ltd., Nanjing, China) was used to detect influenza A/B virus antigens in nasal swab samples.

### 2.6 Statistical analysis

Data were analyzed using SPSS 26.0 software. Count data were expressed as percentages. A *p*‐value of less than 0.05 was considered statistically significant.

## 3 RESULTS

### 3.1 Respiratory pathogens and infection rates

**FIGURE 1:**
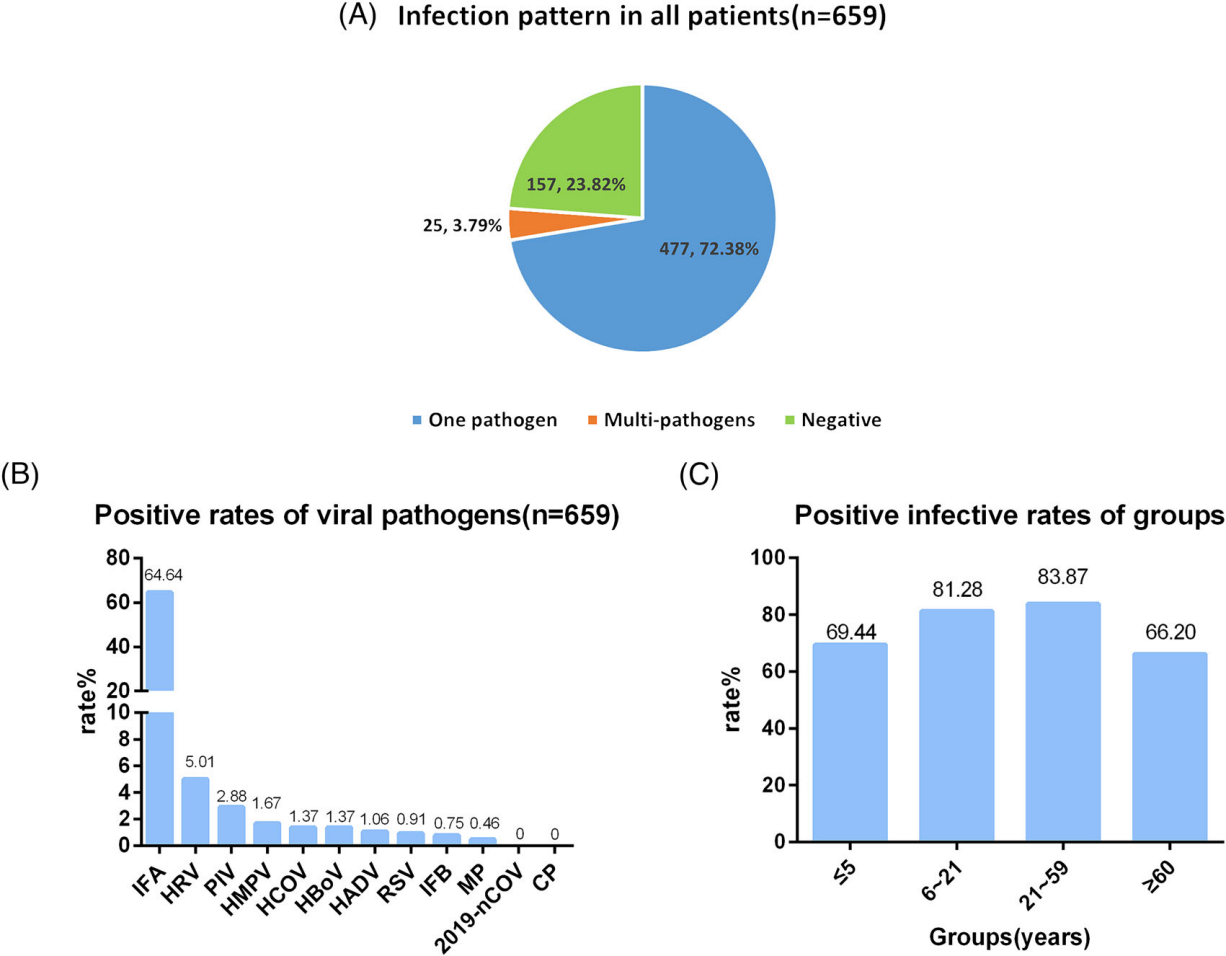
Respiratory pathogens and infection rates. (A) Infection pattern and infection rates in all patients; (B) positive rates of viral pathogens; (C) positive rates of viral pathogens in all groups.

**FIGURE 2:**
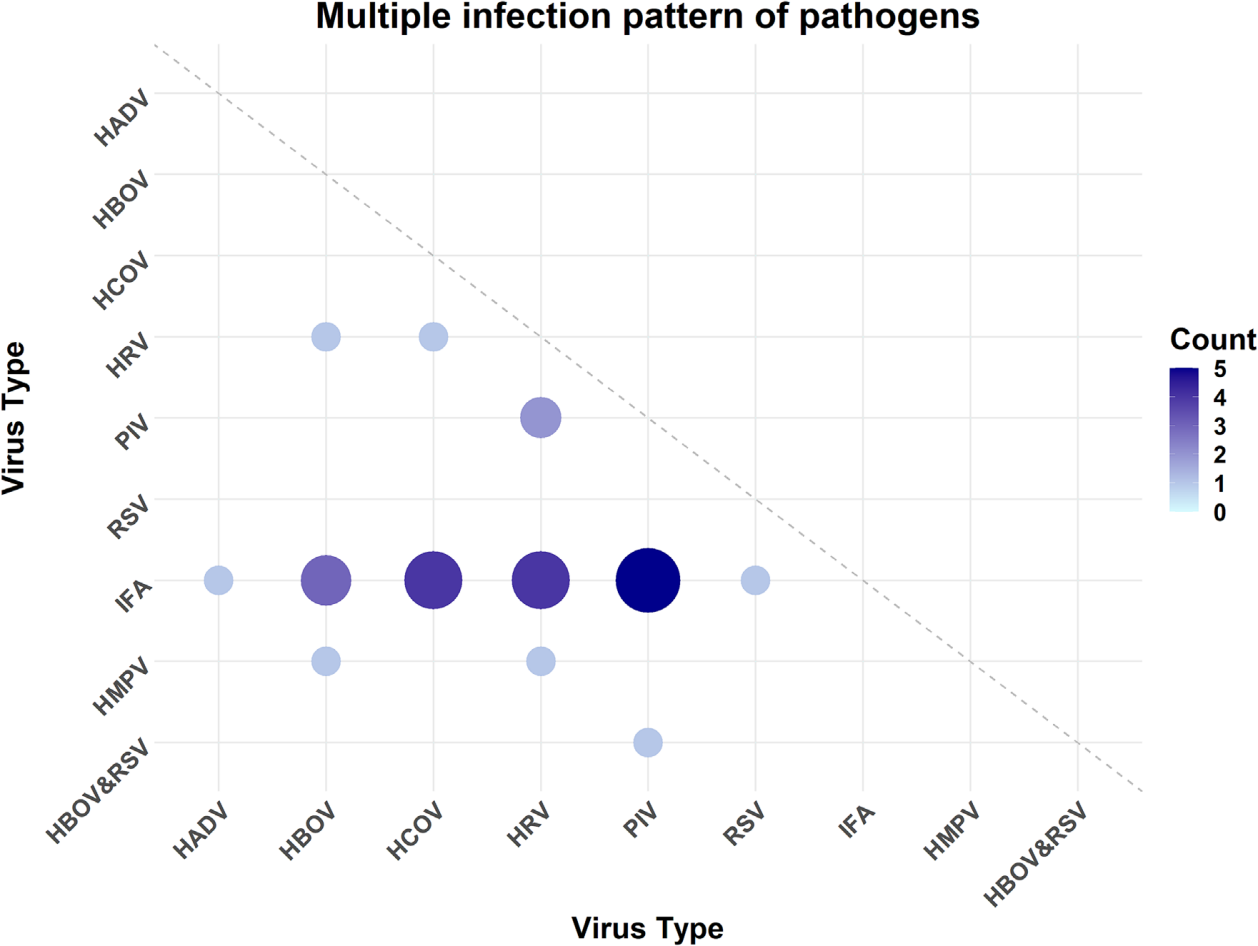
The multiple infection patterns of pathogens.

Of the 659 samples, 330 (50.08%) were from male cases and 329 (49.92%) were from female cases. The age distribution was as follows: 144 cases (21.85%) were aged ≤5 years, 187 cases (28.37%) were aged 6–21 years, 186 cases (28.23%) were aged 22–59 years, and 142 cases (21.55%) were aged ≥60 years. The overall infection rate was 76.18%, with 72.38% single infections and 3.79% multiple infections. No pathogens were detected in 157 cases (23.82%). Among the infected cases, influenza A virus had the highest infection rate at 64.64%, followed by human rhinovirus at 5.01% and parainfluenza virus at 2.88% (Figure 1B). Both the 6–21 and 22–59 age groups had higher infection rates (≥80%) compared with the other age groups (Figure 1C). A total of 25 cases had at least two pathogens detected, accounting for 3.79% of the total samples (25/659). The most common co‐infection was influenza A virus with parainfluenza virus, observed in five cases, followed by co‐infections of influenza A virus with human coronavirus and human rhinovirus, each found in four cases (Figure 2). The patterns of multiple infections are shown in Figure 2.

### 3.2 Comparison of pathogen spectrum among different age groups

**FIGURE 3:**
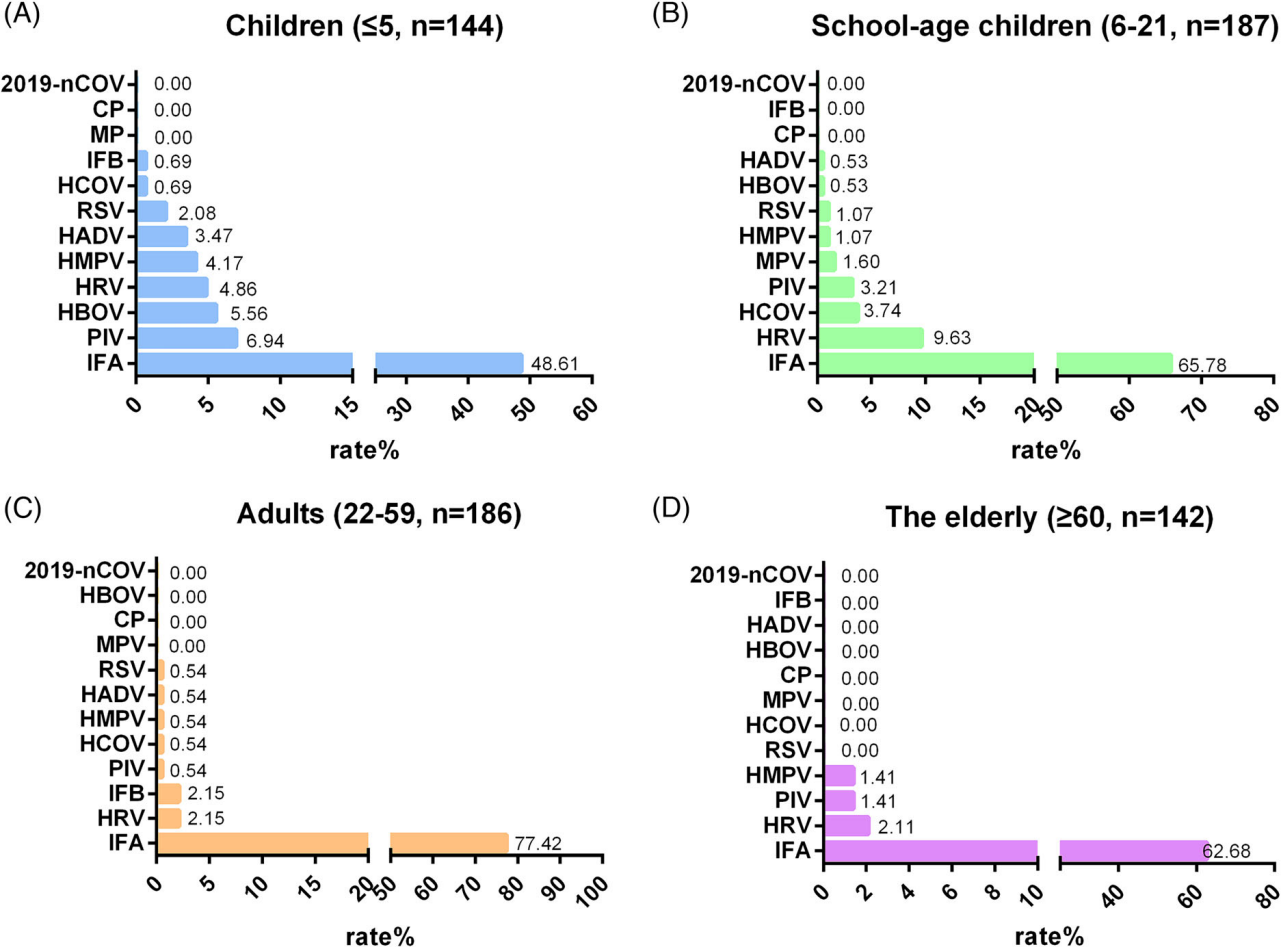
Pathogen spectrum among different age groups. (A) Children; (B) school‐age children; (C) adults; (D) elderly.

Infection rates were compared among children aged ≤5 years, adolescents aged 6–21 years, adults aged 22–59 years, and the elderly aged ≥60 years (Figure 3). Besides the ubiquitous presence of influenza A across all age groups, children aged ≤5 years had high infection rates of parainfluenza and bocavirus. Adolescents aged 6–21 years had high rates of human rhinovirus and coronavirus, adults aged 22–59 years had high rates of human rhinovirus and influenza B, and the elderly aged ≥60 years had high rates of human rhinovirus and parainfluenza virus.

### 3.3 Age and sex distribution of patients infected with influenza A

**FIGURE 4:**
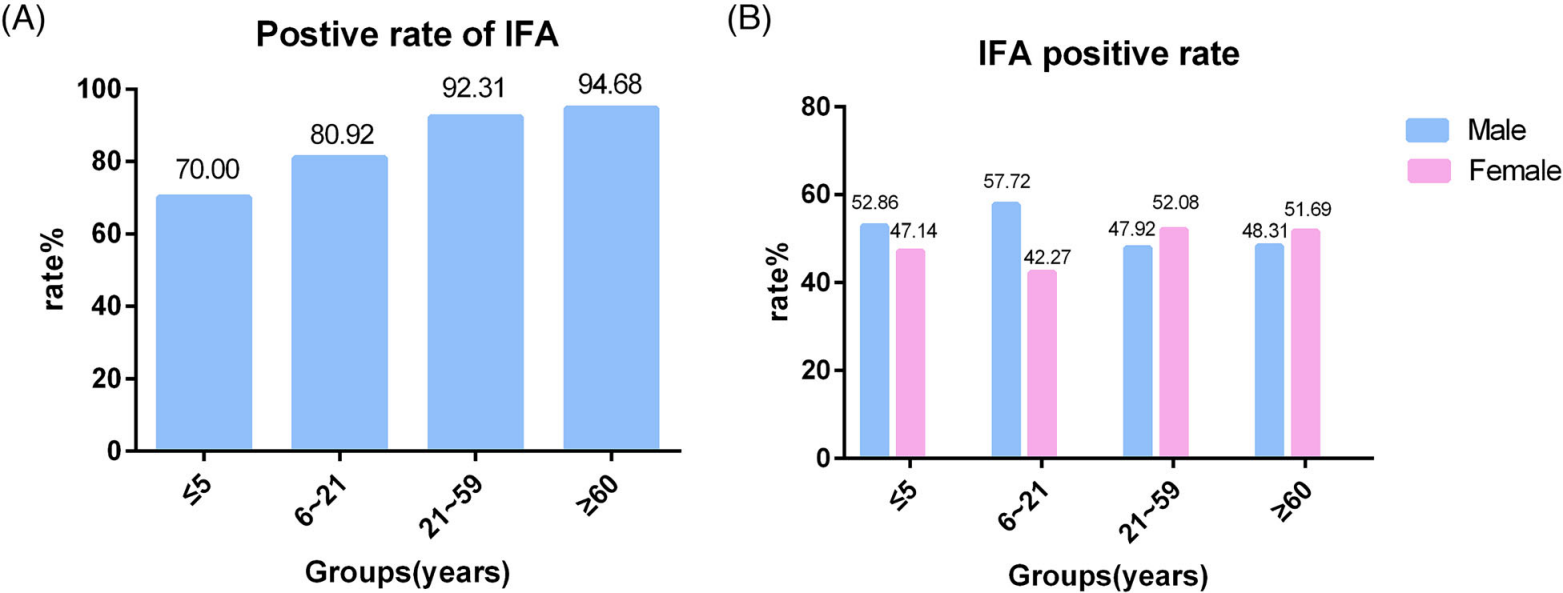
Age and sex distribution of patients infected with influenza A. (A) Age distribution; (B) sex distribution. IFA, influenza A.

No significant difference in the rate of influenza A infection was observed among the four age groups (*p* > 0.05, Figure 4). The influenza A infection rate was highest in the age of ≥60 years group and lowest in the age of ≤5 years group. Males under 21 years of age had a higher rate of influenza A infection than females, whereas the reverse was true for those over 21 years of age.

### 3.4 Comparison of antigen testing and nucleic acid testing results

**FIGURE 5:**
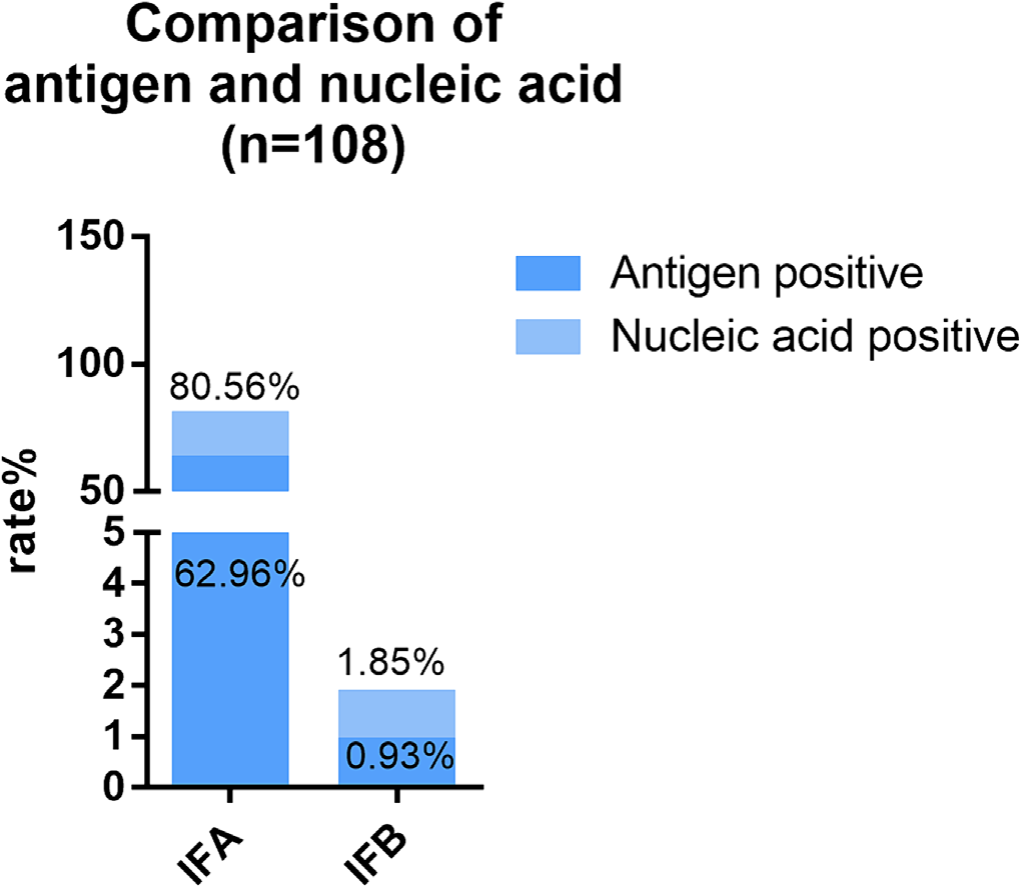
Comparison of antigen testing and MPA results.

The test results of the influenza virus antigen and nucleic acid were compared in 108 randomly selected samples (Figure 5). Of the 87 cases (80.56) with positive nucleic acid results for influenza A, 68 (62.96%) sample antigens were positive. Of the two cases (1.85%) with positive nucleic acid results for influenza B, one sample antigen was negative.

### 3.5 Comparison of MPA PCR and first‐generation sequencing results

**FIGURE 6:**
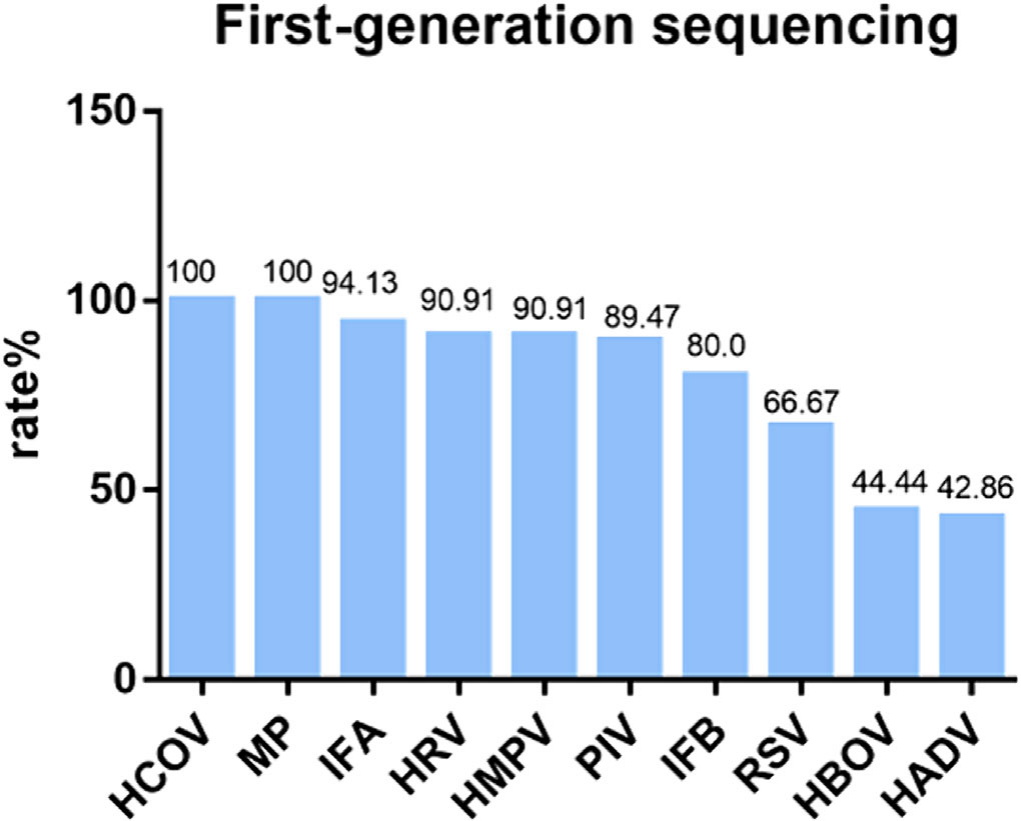
Comparison of MPA and first‐generation sequencing results. Among the positive samples detected by multiplex probe amplification (MPA) PCR, the consistency rate detected by first‐generation sequencing.

The consistency between the results of MPA PCR and first‐generation sequencing for seven pathogens was over 80%, indicating good agreement (Figure 6).

## 4 DISCUSSION

During this influenza season, our hospital had an overall infection rate of 76.18%, composed mainly of single‐heavy infections at 72.38%. Influenza A recorded the highest rate at 64.64%. The pattern also included mixed infections, accounting for 3.79%, most frequently involving influenza A combined with parainfluenza. Across different age groups, the infection pattern consistently showed a high positive rate for influenza A. Previous epidemiological data indicate that Guangzhou experienced influenza outbreaks from 2017 to 2019.^16^, ^17^ COVID‐19 has been shown to alter the epidemiological characteristics of respiratory viruses.^18^ In 2020, COVID‐19 preventive measures contributed to a sharp decline in confirmed influenza cases in Guangzhou starting in February, with some months showing over a 99% reduction compared with the same period in 2019.^15^ Conversely, since June 2022, data from the National Influenza Centre of China indicate a continuous rise in positive tests for influenza. This led the Centers for Disease Control (CDC) in seven southern Chinese provinces to issue influenza warnings for a high‐incidence period dominated by the H3N2 subtype. Our results align with these data, suggesting an ongoing influenza outbreak in Guangzhou as of June. Compared with other influenza viruses, influenza A is more infectious; rapid identification can minimize drug overuse and allow for targeted treatment.^19^

Before the COVID‐19 outbreak, a report by China's CDC indicated that respiratory syncytial virus and human rhinovirus were the primary pathogens in ARI patients under 5 years old in China, followed by parainfluenza and influenza viruses.^20^ Post‐COVID‐19 studies show significant shifts in the ARI pathogenic spectrum, including increased detection rates of metapneumovirus, parainfluenza virus, respiratory syncytial virus, and bocavirus.^21^ Our data also reveal that alongside the widespread prevalence of influenza A, human rhinovirus, parainfluenza virus, bocavirus, coronavirus, and influenza B also vary in prevalence across age groups.

In addition to changes in the pathogenic spectrum, mixed infections are of concern. The CDC in China showed that mixed infections can significantly increase the rate of serious illness in ARI patients.^20^ The most common co‐infection in all age groups was a dual infection between *Streptococcus pneumoniae*, *Mycoplasma pneumoniae*, *Haemophilus influenzae*, and human rhinovirus. In our study, mixed infections accounted for 3.79%, primarily comprising influenza A combined with other viruses.

With the normalization of COVID‐19 epidemics, the rate of COVID‐19 increases, and COVID‐19 can be superimposed on other pathogenic infections.^22^, ^23^ In 2021, global COVID‐19 co‐infection statistic data showed a 0%–49.5% co‐infection rate of COVID‐19 with other common viruses across China, and the most common viruses co‐infected with COVID‐19 were influenza, syncytial virus, human rhinovirus, and adenovirus.^24^ The risk is increased by the superimposition of other pathogens on COVID‐19. A British study shows that compared with only those infected with COVID‐19, adults infected with COVID‐19 and influenza virus at the same time are 4.14 times more likely to need ventilator treatment and 2.35 times more likely to die.^25^ A study in Wuhan shows that 5.8% of 8274 COVID‐positive patients and 18.4% of non‐COVID‐19 infected patients have infections with other pathogens.^26^ In conclusion, with the normalization of epidemics, the superposition of COVID‐19 and influenza or other pathogens may become increasingly common, further increasing uncertainty and complexity, and the identification of COVID‐19 from other respiratory pathogens is particularly necessary.

There are as many as 20–30 common pathogens of respiratory tract infections, and multiple methods are available for their detection.^27^ Among these, nucleic acid detection techniques enjoy widespread use because of their high sensitivity and specificity.^28^ In our study, an MPA technology kit was employed, capable of detecting 12 targets in a single tube. The technology leverages molecular beacon probes and melting curves, allowing a single fluorescent channel to identify four to six targets.^29^ When comparing nucleic acid detection results of positive samples against Sanger sequencing, over 80% showed concordance, whereas discordant samples predominantly had viral copy numbers near the threshold. This discrepancy may stem from the differing sensitivities between the two techniques. The higher sensitivity of the PCR method allows for the detection of samples with low copy‐size viruses. Notably, adenovirus and HBOV showed less consistency, at 42.86% and 44.44%, respectively. The small number of positive samples for these pathogens—fewer than 10—could explain the low concordance rate. Additionally, in a comparison between nucleic acid and antigen test results for 108 samples, 17.6% of those testing positive for influenza A through nucleic acid tests tested negative in the antigen test. Also, one of two nucleic acid‐positive samples for influenza B tested negative in the antigen test, further illustrating that nucleic acid tests are significantly more sensitive than antigen tests.

## 5 CONCLUSION

In conclusion, the Guangzhou region experienced an influenza outbreak in June 2022. The pathogenic spectrum has evolved before and after the COVID‐19 outbreak, influenced by factors such as the epidemic, geography, and climate. More intensive epidemiological surveillance is necessary for comprehensive data collection. As the epidemic becomes more regular, the overlap between COVID‐19 and influenza is likely to increase, necessitating differentiation from other respiratory pathogens. MPA testing, being both efficient and consistent with sequencing, could be the first choice for screening respiratory infections.

## AUTHOR CONTRIBUTIONS

Yuezhi Deng conceived and designed the study, performed the experiments, analyzed the data, and wrote the manuscript. Weihong Lin contributed to the study design, provided critical feedback on the data analysis, and revised the manuscript. Yongping Lin supplied the necessary resources and materials for the study and assisted in data collection.

## CONFLICT OF INTEREST STATEMENT

The authors declared that there are no conflicts of interest in this work.

## ETHICS STATEMENT

This study was approved by the Ethics Committee of the First Affiliated Hospital of Guangzhou Medical University. Consent of publication has been obtained from the patients.

## Supporting information


**Table S1.** **Sanger sequencing primers.** IFA: Influenza A virus; IFB: Influenza B virus; RSV: Respiratory syncytial virus; HADV: Human Adenovirus; HMPV: Human Metapneumovirus; HBOV: Human Boca virus; MP: Mycoplasma pneumonia; CP: Chlamydia pneumonia; HRV: Human Rhinovirus; 229E: Coronavirus subtype 229E; NL63:Coronavirus subtype NL63
OC43: Coronavirus subtype OC43; HKU1: Coronavirus subtype HKU1; PIV1:Parainfluenza virus type 1; PIV2: Parainfluenza virus type 2; PIV3: Parainfluenza virus type 13; PIV4: Parainfluenza virus type 4; ORF1Ab: ORF1ab gene of COVID‐19; N: N gene of COVID‐19.

## Data Availability

The data that support the findings of this study are available from the corresponding author upon reasonable request.
